# Interfacial Failure Cascade and Modulation Strategies for Layered Oxide Cathodes in Sodium-Ion Batteries

**DOI:** 10.3390/molecules31193551

**Published:** 2026-10-05

**Authors:** Yong-Gang Sun, Hao-Jie Fan, Yi-Han Zhang, Jian Xiong, Jin-Yi Ding, Jun-Yu Zhao, Jia-Feng Ru, Meng-Shuang Lei, Chen Cheng, Xi-Jie Lin

**Affiliations:** 1 School of Chemistry and Chemical Engineering, Yancheng Institute of Technology, Yancheng 224051, China; fhj18012096918@163.com (H.-J.F.); ruarua0505@163.com (Y.-H.Z.); xiongjian1917@163.com (J.X.); djy11062026@163.com (J.-Y.D.); zjy19551502383@163.com (J.-Y.Z.); jaru_feng@163.com (J.-F.R.); leimengshuang_2026@163.com (M.-S.L.); chengchen103985@163.com (C.C.); 2 Jiangsu Key Laboratory of Coastal Photovoltaic Technology and System, Yancheng Institute of Technology, Yancheng 224051, China; 3 School of Chemistry and Pharmaceutical Sciences, Guangxi Normal University, Guilin 541004, China

**Keywords:** sodium-ion batteries, layered oxide cathodes, interfacial failure, failure cascade, chemo-mechanical coupling, surface coating, interface engineering

## Abstract

Layered transition-metal oxides (Na_x_TMO_2_) are among the most promising cathode candidates for sodium-ion batteries, yet their interfacial degradation under high voltage, deep desodiation, and humid environments remains the central bottleneck to commercialization. Here we argue that interfacial failure of layered cathodes is not a collection of isolated events but a coupled failure cascade in which chemical, structural, and mechanical factors amplify one another along a causal chain—surface residual alkali and oxygen release, heterogeneous phase transitions and surface reconstruction, stress concentration and crack propagation, and finally electrolyte infiltration with self-catalyzed formation of fresh interfaces. Starting from this cascade, we extract the corresponding modulation targets and, organized along the atomic–nano–micro length scale, survey single-axis strategies that include pillar-ion pinning, in situ conversion of residual alkali, gradient and epitaxial coatings, intergrown heterointerfaces, multifunctional coatings, and concentration-gradient particles. We then synthesize cross-scale synergistic paradigms—coating-plus-doping cascades, mechano-electrochemical coupling, dynamically stable interfaces under wide-temperature and high-voltage operation, and cathode–electrolyte interphase (CEI) engineering—and show that interface modulation is shifting from passive isolation toward active regulation. We close by distilling actionable design principles and outlining prospects for responsive interfaces and rational design.

## 1. Introduction: The Interfacial Cascade of Layered Cathodes—A Domino Effect of Failure

Sodium-ion batteries (SIBs), owing to the abundance and low cost of sodium resources and a working principle analogous to lithium-ion batteries, are widely regarded as one of the most competitive complementary technologies for grid-scale energy storage and low-speed electric vehicles [1,2,3]. Unlike lithium, sodium does not alloy with aluminum, so aluminum current collectors can be used on both electrodes, and together with hard-carbon anodes and earth-abundant cathode metals, this positions SIBs as a near-term, cost-driven alternative to lithium iron phosphate (LFP) chemistry for stationary storage [2,3,4].

Among candidate cathodes, layered transition-metal oxides Na_x_TMO_2_ (TM = Mn, Ni, Fe, Co, etc.) have advanced to become among the most commercially developed by virtue of their high theoretical capacity, simple synthesis, and compatibility with lithium-battery production lines [1,5,6]. Following the structural classification of Delmas and co-workers, these materials are divided into O3-, P2-, and P3-type stacking according to the Na coordination environment (octahedral versus prismatic) and the oxygen packing [7]. P2 cathodes offer direct prismatic Na^+^ diffusion channels and superior rate capability but contain less Na (x ≤ 0.8), whereas O3 cathodes store more Na (0.8 < x ≤ 1) and pair naturally with Na-free hard-carbon anodes but suffer slower kinetics, complex phase transitions, and greater air sensitivity [5,6,8]. Earth-abundant P2-Na_2/3_[Fe_1/2_Mn_1/2_]O_2_, reported as early as 2012, exemplified the cost potential of this family [9]. These attributes have propelled layered oxides toward commercial viability, particularly for grid-scale energy storage where cost and sustainability are paramount.

Yet, the very same structural flexibility that enables reversible sodium intercalation becomes the Achilles’ heel of these materials when they are pushed toward high energy density. Electrochemical extraction of sodium beyond a critical threshold triggers a cascade of interconnected failure processes that emanate from—and feed back into—the interface between the oxide particle and the electrolyte. Deep desodiation induces irreversible phase transitions (O3 → P3 → OP2 or P2 → O2), causing abrupt lattice volume changes and anisotropic strain [10,11,12]. Simultaneously, the highly oxidized surface (Ni^4+^, Fe^4+^, and reactive lattice oxygen) catalyzes electrolyte decomposition, while the acidic species generated (HF) corrode the surface and dissolve transition metals [13,14]. These chemical and structural instabilities are not isolated events; they conspire with mechanical stresses to create a self-amplifying “interfacial vortex” that rapidly degrades capacity, rate capability, and safety.

The conventional narrative often dissects cathode degradation into five modes: surface reconstruction, transition metal dissolution, electrolyte oxidation, oxygen release, and particle cracking [15,16,17,18,19]. However, mounting evidence from operando spectroscopy, high-resolution microscopy, and chemomechanical modeling reveals that these processes are deeply entangled. Surface rock salt formation is not merely a structural passivation layer; it disrupts the local Na percolation network, exacerbates heterogeneous state of charge, and amplifies internal stress gradients [20,21]. The resulting microcracks, in turn, expose fresh surfaces to the electrolyte, accelerating dissolution and gas evolution [22]. In this way, a minor interfacial perturbation can trigger a domino-like collapse that propagates from the particle surface to its interior and ultimately to the entire composite electrode.

Several recent reviews have addressed interface modification and chemo-mechanical degradation of sodium layered oxides [17,18,22], and the coupled nature of these processes is itself well documented [23]. The distinctive contribution of the present review is threefold. First, the degradation pathways are organized into an explicit causal–feedback network in which chemical, structural, and mechanical processes are connected by named, directional couplings (e.g., transition-metal dissolution weakens grain boundaries; phase-transition stress nucleates cracks; cracks expose fresh surface that re-accelerates interfacial reactions), rather than merely listed in parallel. Second, every modulation strategy is mapped onto the specific link(s) and length scale it interrupts, making explicit why superficially similar coatings differ and which failure mode each strategy can and cannot address. Third, the field’s trajectory is framed as a shift from passive protection to active regulation.

This review develops the thesis in four steps: dissecting the failure chain, extracting modulation targets, distilling design principles, and moving toward synergy and practical deployment. We first construct a chemo-structural-mechanical coupling diagram of interfacial failure and clarify the recursive, self-catalyzing relationships among failure modes; we then re-organize single-axis interface-reconstruction strategies along the length-scale axis; subsequently, we focus on multifunctional synergistic interfaces and CEI engineering that transcend individual strategies, arguing that modulation is shifting from passive isolation to active regulation; and finally, we distill actionable design principles and outline future directions. Throughout, “interface” is taken to encompass four levels—particle surfaces, grain boundaries, the electrode–electrolyte interface (CEI), and the cross-electrode interface—whose spatial origins of failure map one-to-one onto the modulation length scale, which is what motivates the cross-scale narrative adopted here. By reframing interfacial engineering as the art of disrupting a failure cascade, this review aims to provide both a coherent understanding of current progress and a conceptual toolkit for designing the next generation of robust, high-energy sodium-ion battery cathodes.

## 2. A Multi-Field Coupling Diagram of Interfacial Failure: Chemical, Structural, and Mechanical Frontiers

Rather than listing failure modes side by side, this chapter builds a multi-field coupling diagram. The chemical, structural, and mechanical frontiers each define a trigger mechanism, but what proves fatal is their recursive interaction: once a single failure is initiated, it amplifies along the causal chain into a positive-feedback loop. Two terminological notes are in order. “Frontier” is used deliberately rather than “mechanism” because each of the three categories is not a single process but a family of coupled processes that mark the boundary beyond which the material cannot return to its pristine state; and the three frontiers are presented sequentially only for clarity—in operation they act concurrently rather than in sequence.

### 2.1. Chemical Frontiers

Which process acts as the initiating event is system- and condition-dependent; chemical failure is a common but not universal trigger. The first trigger is the catalytic network of electrolyte oxidative decomposition: at high states of charge, highly oxidized TM ions (Ni^4+^, Fe^4+^) and reactive surface oxygen species catalyze the oxidation of carbonate electrolytes, and the resulting high-impedance byproducts accumulate continuously on the surface. Systematic X-ray photoelectron spectroscopy on P2/O3-Na_2/3_Ni_1/3_Mn_2/3_O_2_ revealed that the outermost interface is enriched in a suite of fluorides (polyvinylidene fluoride, NaF, NiF_2_, MnF_2_), generated by the reaction of HF with the electrode; in parallel, CO_2_ release—originating from electrolyte or residual-carbonate decomposition—begins near 3.6 V and rises linearly to a maximum at the end of charge [6]. Mu et al. used aberration-corrected STEM combined with EELS to observe directly how the O3-NaNi_1/3_Fe_1/3_Mn_1/3_O_2_ surface is progressively reconstructed from a layered phase into disordered rock-salt/spinel phases, accompanied by Mn reduction, lattice-oxygen loss, and nanocracks along Na^+^ diffusion channels [24]. A second trigger is the self-sustaining cycle of TM dissolution and acid corrosion: trace water in NaPF_6_/FEC electrolytes generates HF, which attacks TM–O bonds and dissolves Mn, Ni, and Fe, and the dissolved ions in turn catalyze further electrolyte decomposition [5,6,18]. Low-valence Mn species are especially damaging because their Jahn–Teller activity and dissolution create lattice defects that further destabilize neighboring TM sites [6].

**Figure 1:**
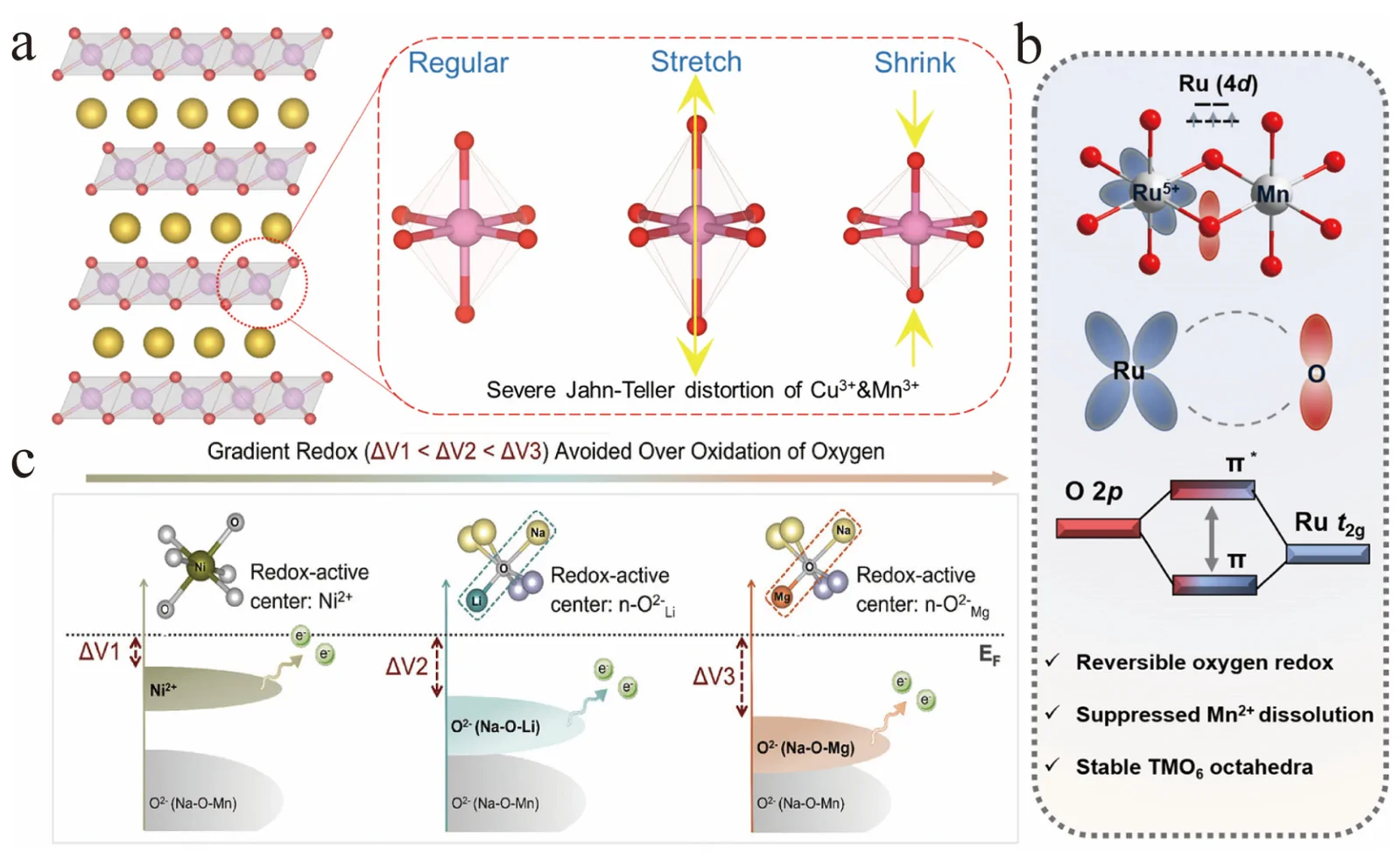
(**a**) Schematic diagram of octahedral phase transformation and mechanism diagram of crystal deformation suppression by Mg/Cu co-doping [29]. (**b**) Schematic illustration of the role of π-type interaction in Na_0.6_Li_0.2_Mn_0.7_Ru_0.1_O_2_ [27]. (**c**) Schematic of three redox centers (Ni^2+^, n-O^2−^_Li_ and n-O^2−^_Mg_) in the O-Mn-LNM sample. The gradient redox activity thermodynamically avoids the over-oxidation of oxygen ions [30]. Reproduced/Adapted with permission from the cited references.

The third is irreversible lattice-oxygen release. Above roughly 4.0–4.2 V, anionic redox is activated and non-bonding O 2p states are oxidized to peroxide/superoxide species or even gaseous O_2_, propagating from surface oxygen vacancies into the bulk and ultimately collapsing the structure [25,26,27,28,29]. Several distinct mechanisms have been proposed to render this redox reversible. In P2-Na_2/3_Mn_7/12_Mg_1/4_Fe_1/6_O_2_, Fe acts as a redox mediator that captures electrons from lattice oxygen during charge and donates them back during discharge, raising the reversibility of lattice oxygen from 75% to 99% [25]. In O3-type Li–Mg co-substituted Na_1-x_Li_1/3-x_Mg_x_Mn_2/3_O_2_, interlayer Mg^2+^ migration into tetrahedral interstices pins the lattice, inhibits Mn intralayer migration and vacancy clustering, and lifts capacity retention from 36.5% to 85.5% over 120 cycles [28]. Cu/Mg co-doping in P3-Na_0.67_Mn_0.9_Mg_0.05_Cu_0.05_O_2_ triggers an “adaptive lattice breathing” (Figure 1a) in which Cu oxidation and Mg-driven oxygen-framework expansion compensate each other, yielding 258.1 mAh g^−1^ [29]. Ru^4+/5+^ incorporation strengthens the TM–O π-type interaction (Figure 1b) to stabilize anionic redox in Na_0.6_Li_0.2_Mn_0.7_Ru_0.1_O_2_ [27], and gradient de-clustered anionic redox has been shown to suppress O_2_ formation (Figure 1c) to undetectable levels at 4.5 V [30]. A common thread across these examples is the manipulation of TM–O covalency and of the local oxygen coordination: modulating TM–O covalency was shown early on to enhance the reversibility of lattice-oxygen redox [31], strong covalent bonds were used to constrain interlayer slipping in P2 oxides with oxygen redox [32], and whole-voltage-range oxygen redox was demonstrated in P2 cathodes [33].

Air degradation is likewise chemical in origin. P2 oxides degrade in humid air predominantly through Na^+^/H^+^ exchange that deposits NaOH/Na_2_CO_3_ on the surface, whereas O3 oxides, with their higher Na content, are even more air-sensitive [5,34]. Zuo et al. established that CO_2_ does not intercalate into the layered framework (as shown by surface-sensitive TOF-SIMS), that Na^+^/H^+^ exchange is the dominant charge-compensation pathway, and that air stability can be ranked empirically by the first-cycle redox potential—materials with high-potential couples (Ni^2+/4+^, Cu^2+/3+^, O^2−/n−^) being markedly more stable than those with V, Mn, Co, or Fe redox [34]. Two aspects of this chemical frontier deserve emphasis. The first is its catalytic character: the failure is not a stoichiometric corrosion but a self-propagating cycle, because both the dissolved TM ions and the reactive surface oxygen that they expose are themselves catalysts for further electrolyte decomposition [5,6,18]. The second is its connection to air stability: the same residual-alkali species that form on exposure to humid air are precisely the species that, once the cell is assembled, feed the HF-driven corrosion cycle, so air sensitivity and cycling instability share a common surface origin [16,34].

### 2.2. Structural Frontiers

**Figure 2:**
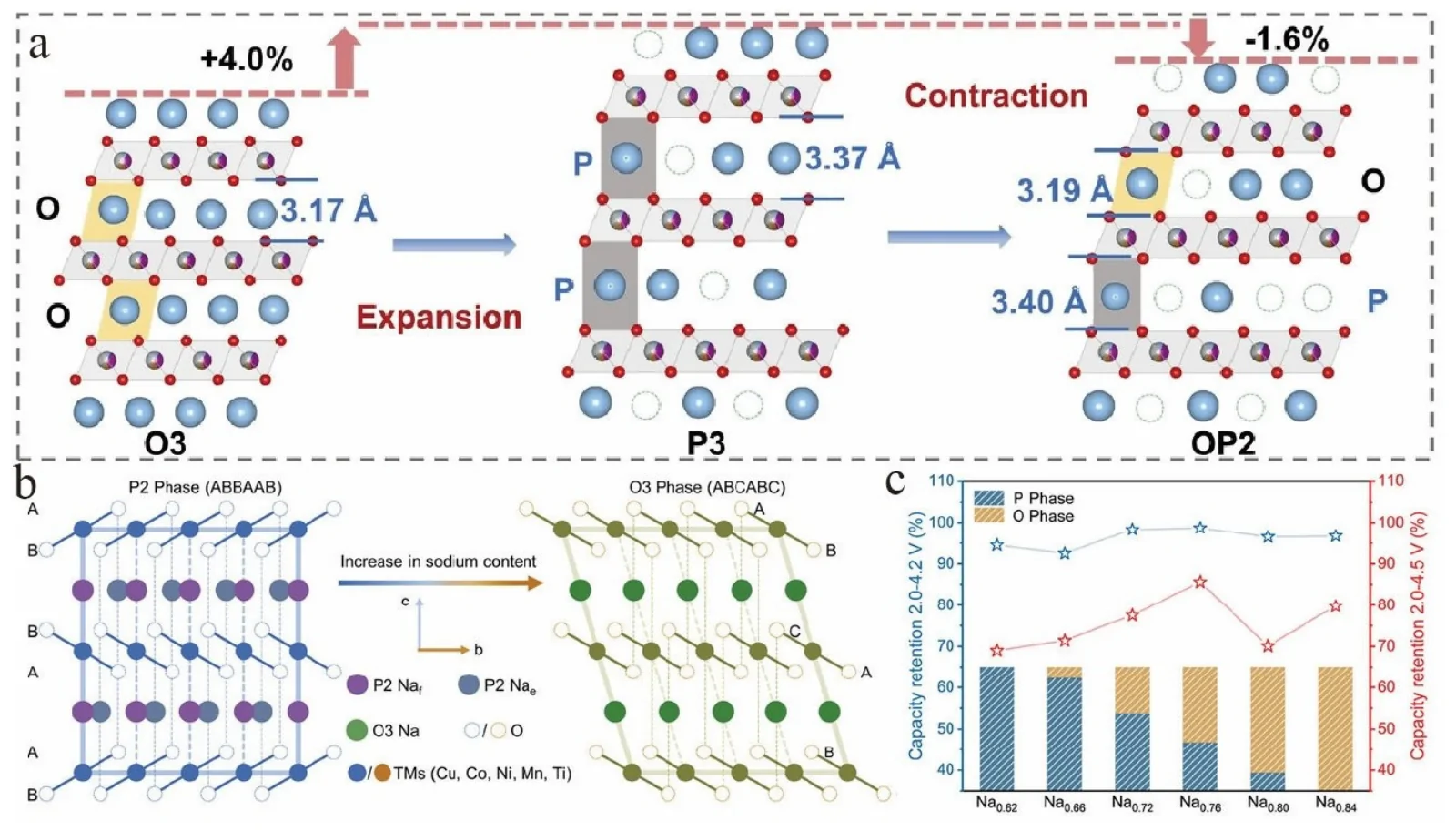
(**a**) Schematic illustrations of the structural change in Na_0.9_Ni_0.32_Zn_0.08_Fe_0.1_Mn_0.3_Ti_0.2_O_2_ during Na^+^ extraction [36]. (**b**) Schematic illustration of phase transition from P2 phase to O3 phase with increase in Na- stoichiometries. (**c**) Capacity retention rate under different voltage ranges [37]. Reproduced/Adapted with permission from the cited references.

Structural failure provides the skeleton of the cascade. Heterogeneous phase transitions—O3 → P3 → O3′, P2 → O2/OP4 and related sequences—involve interlayer gliding and TMO_>2_-layer distortion, and the lattice mismatch between coexisting phases generates localized stress within particles [1,5,8,19]. Operando XRD on O3-NaNi_1/3_Fe_1/3_Mn_1/3_O_2_ has resolved the O3→P3→O3′ sequence and the accompanying c-axis evolution that underlies the irreversible P3–P3′ transition at high voltage [35]. In P2-Na_0.67_Li_0.1_Fe_0.37_Mn_0.53_O_2_, excessive desodiation additionally nucleates O-type stacking faults that form an intergrowth of O- and P-type layers, driving out-of-lattice Li migration and irreversible oxygen loss [26]. Beyond the phase-sequence details, the structural consequence can also be engineered away: an O3-type oxide designed for negative lattice expansion during cycling suppresses the volume change (Figure 2a) and particle degradation, markedly improving long-term stability [36], and a systematic study of binary-phase cathodes (Figure 2b,c) reveals that the interphase between coexisting phases, rather than their mere coexistence, governs structural stability [37].

Meanwhile, under electrolyte attack and oxygen loss, the particle surface reconstructs into electrochemically inert rock-salt (NiO-like) and disordered spinel phases; these layers form randomly along (003) planes with abundant dislocations and severely block Na^+^/electron transport [6,24]. The spinel phase is often an intermediate stage on the way to the rock-salt end state, and its heterogeneous, anisotropic distribution along Na^+^ diffusion channels further aggravates the transport penalty [24]. Cation mixing and the spreading of “broken pillars” in the Na layer add a final instability: TM ions (especially Fe and Ni) migrate into the Na layer, both occupying Na sites and vacating the TM sites that otherwise support the interlayer, weakening the framework and accelerating gliding. At still higher voltages, the collapse of the Na layer can nucleate an OP2 phase [18,19]. Both high-entropy cathodes that stabilize a reversible P3–OP2–P3 transition and coatings that exploit a P3–OP2 intergrowth have been used to blunt this otherwise detrimental transformation [38,39]. A further, often under-appreciated aspect of the structural frontier is the heterogeneity of surface reconstruction. Because the rock-salt and spinel phases nucleate preferentially along exposed (003) planes and Na^+^ diffusion channels, the reconstructed layer is neither uniform nor self-passivating: it blocks transport where it is thickest while leaving adjacent regions exposed to further attack [6,24]. This heterogeneity is why a thick but discontinuous coating can fail—the degradation simply relocates to the unprotected regions—and it motivates the emphasis on conformal, lattice-matched coverage [40,41].

### 2.3. Mechanical Frontiers

**Figure 3:**
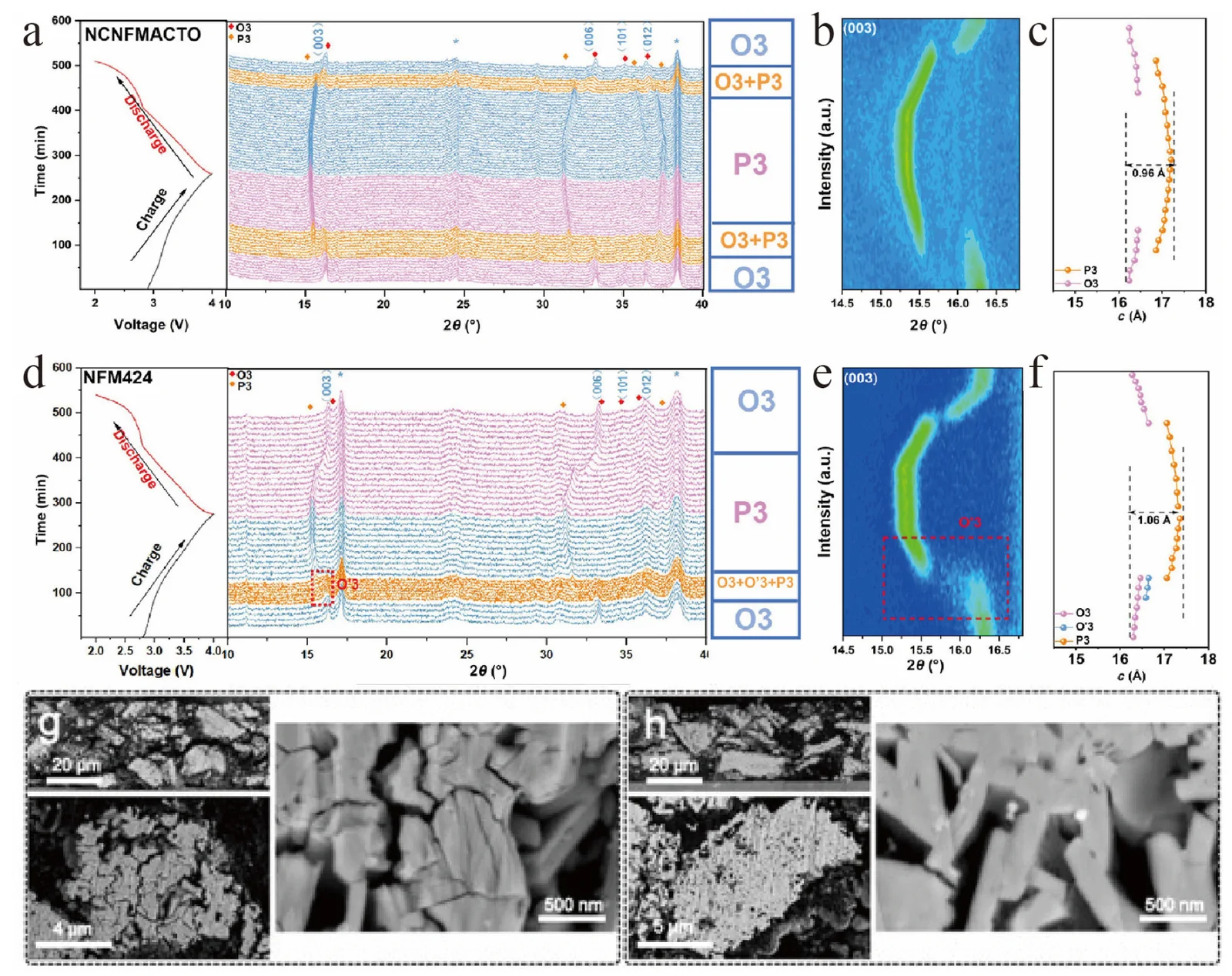
In situ X-ray diffraction analysis and post-cycling structural examination: in situ XRD with intensity contour maps of Na_0.96_Ca_0.02_Ni_0.27_Fe_0.2_Mn_0.35_Al_0.02_Cu_0.1_Ti_0.05_O_2_ (NCNFMACTO) (**a**,**b**) and NaNi_0.4_Fe_0.2_Mn_0.4_O_2_ (NFM424) (**d**,**e**); (**c**,**f**) the calculated lattice parameters (*c*) evolution during the charging process [43]. SEM images of (**g**) NaNi_0.4_Fe_0.1_Mn_0.4_Mg_0.05_Ti_0.05_O_2_ (NFMMT) and (**h**) NFMMT/NaCaPO_4_ after 200 cycles [41]. Reproduced/Adapted with permission from the cited references.

Mechanical failure amplifies the cascade. During (de)sodiation, the anisotropic changes in the a and c lattice parameters produce a non-uniform stress field within secondary particles—compressive at the core, tensile at the surface [41,42]. High-entropy designs can compress the O3–P3–O3 volume strain to as little as 1.1% (Figure 3a–f), removing crack sources at their origin [43]. Chemo-mechanical coupling then drives cracking: TM dissolution weakens intergranular bonding, while phase-transition stress initiates and propagates cracks along grain boundaries and (003) planes, so that chemical degradation and mechanical fracture reinforce each other [6,35,42]. Cross-sectional electron microscopy after cycling routinely shows intragranular cracks and particle exfoliation in unmodified layered cathodes, whereas structurally stabilized or coated particles remain intact [44,45]. The cracks themselves become new interfaces: each fracture exposes fresh reactive surface through which electrolyte penetrates toward the interior, spreading failure from the surface into the bulk in a self-catalyzing “crack–infiltration–new interface–worse failure” loop (Figure 3g,h) [41]. This is the mechanical origin of the domino-like character of layered-cathode failure. Because the a-axis expansion and c-axis contraction are not synchronous, the strain is inherently multiaxial; isotropic particle morphologies and single crystals, which remove grain boundaries, consequently suppress crack nucleation at its source [40,45]. It is worth distinguishing two mechanistically different crack sources that are often conflated. Grain-boundary cracks originate from the anisotropic strain of randomly oriented primary particles, whereas intragranular cracks along (003) planes originate from the c-axis breathing of the layered structure itself [6,40,42]. The two call for different remedies: the former favors isotropic particle morphology and single crystals, the latter favors interlayer pinning and lattice-coherent interfaces that restrain slab gliding [40,45]. Recognizing which source dominates in a given material is therefore a prerequisite for choosing the correct mechanical intervention.

Linking the three frontiers yields a coupled feedback network (rather than a fixed linear sequence): surface residual alkali and reactive oxygen trigger electrolyte decomposition and HF attack (chemical), which drives TM dissolution and lattice-oxygen loss, which in turn drives heterogeneous phase transitions and rock-salt reconstruction (structural); the resulting anisotropic stress concentration nucleates cracks (mechanical), and electrolyte infiltration along these cracks exposes fresh interfaces that further intensify interfacial side reactions and broaden phase-transition stress (returning to chemical/structural) [5,6,42,46]. Once closed, this loop is self-catalyzing and self-amplifying—the physical content of the “interfacial vortex” and “domino effect”. This leads to our overarching principle: an effective intervention should sever a critical weak link of the cascade rather than pile up protection indiscriminately. One consequence of the feedback structure is that interventions are asymmetric in leverage: severing the loop early—for example, at residual-alkali formation or at the first phase transition—can forestall the mechanical failure that would otherwise follow, whereas acting late, once cracks have formed, only slows an already-running process [5,46]. This asymmetry explains the outsized impact of comparatively subtle surface modifications, and it cautions against judging a strategy solely by its final retention figure without asking which link it actually severed [41,47]. A second consequence is diagnostic: because the loop is closed, a single measured symptom—capacity fade, gas evolution, or impedance growth—rarely identifies the initiating cause, which is why the multi-technique approach is indispensable [6,24,44]. In this network, the links demonstrated directly by operando XRD, DEMS, or STEM/EELS (phase sequence, gas evolution, surface reconstruction) are distinguished from couplings inferred by synthesis across material systems.

Because the dominant failure mode differs by chemistry, a strategy optimal for one system cannot be assumed to transfer to another. Ni-rich systems are dominated by slab gliding and Ni migration at high states of charge; Mn-rich systems by Jahn–Teller distortion, Mn^3+^ dissolution, and air sensitivity; Fe-containing systems by Fe^4+^ migration and, in some compositions, Fe-mediated oxygen redox; and oxygen-redox-active systems by lattice-oxygen loss and voltage hysteresis.

## 3. The Design Language of Modulation: Interface Reconstruction Across Atomic, Nano, and Micro Scales

This chapter re-organizes the diverse modification strategies along the length-scale axis. It deliberately treats only single-axis interventions—doping alone, coating alone, or heterointerface construction alone—to establish the correspondence between modulation scale and the failure level it addresses; cross-scale synergy is deferred to Section 4. The underlying logic is that failure has a spatial origin—surface chemistry, near-surface reconstruction, and whole-particle mechanics—so the intervention scale should mirror the failure scale. An important corollary is that no single length scale is privileged: a strategy is not more fundamental simply because it operates at the atomic scale, since a purely chemical fix cannot by itself arrest a mechanical cascade and vice versa [40,42]. The following three subsections should therefore be read as complementary, not competing, tiers.

### 3.1. Sub-Nanometer Doping and Chemical Adsorption (Atomic Scale)

**Figure 4:**
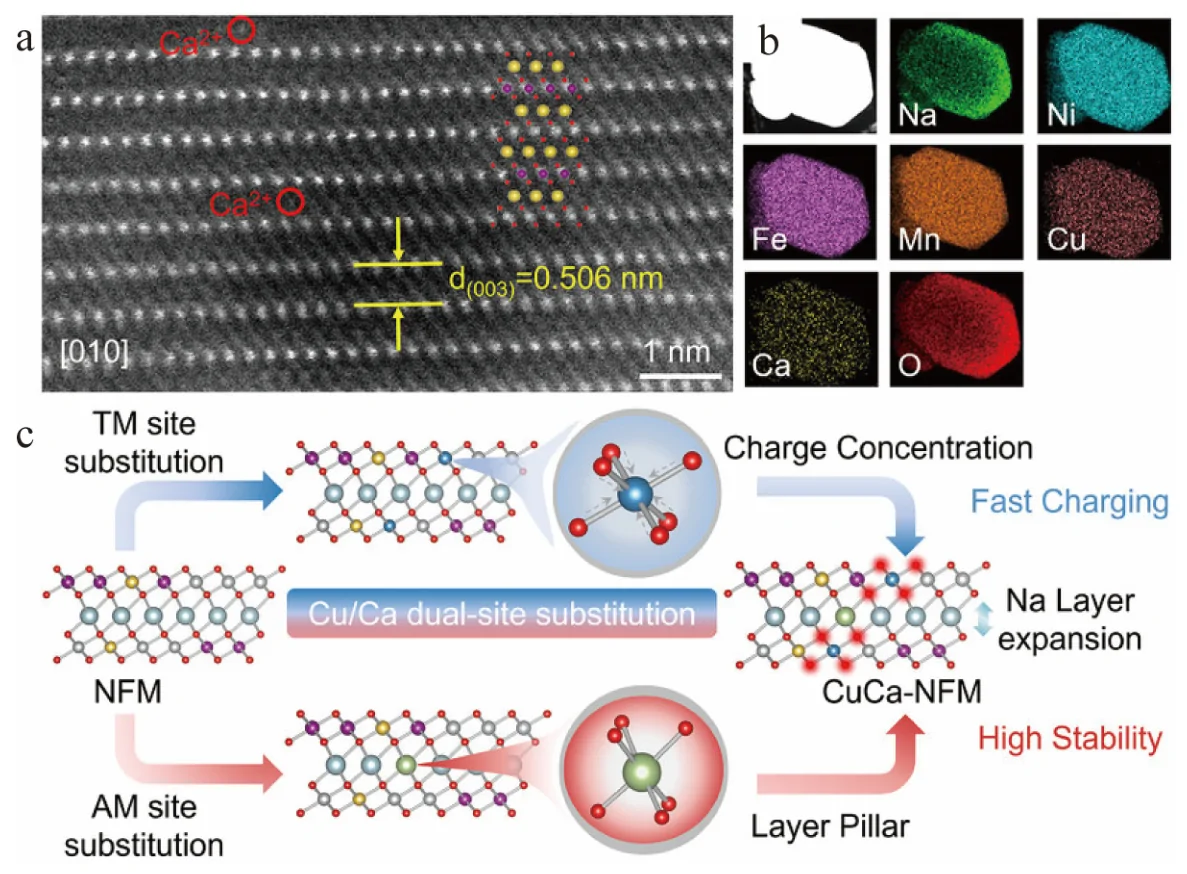
(**a**) HAADF-STEM images of Na_0.98_Ca_0.01_Ni_0.35_Cu_0.05_Fe_0.2_Mn_0.4_O_2_ (CuCa-NFM) from [010] zone-axes. (**b**) EDS elemental mapping and (**c**) schematic diagram for the mechanism of Cu_TM_ and Ca_AM_ dual-site substitution to realize the fast-charging and high-stability characteristics of CuCa-NFM [48]. Reproduced/Adapted with permission from the cited references.

Atomic-scale modulation acts at the front of the cascade, suppressing interlayer gliding and residual-alkali formation. Pillar-ion pinning is one lever: Ca^2+^, whose radius approaches that of Na^+^, preferentially occupies the Na layer and acts as an interlayer pillar that restrains gliding and phase transitions during deep desodiation, whereas Li^+^, Mg^2+^, Al^3+^, and Ti^4+^ enter the TM layer and strengthen TM–O bonds [41,48,49,50]. The concept traces back to Mg doping, which was shown early on to stabilize the P2-Na_2/3_Mn_1-y_Mg_y_O_2_ framework and deliver high-rate performance [51]. More recently, Cu/Ca co-doping (Figure 4a,b) has been used to stabilize both the Na and TM layers simultaneously, enabling fast charging and high stability in O3 cathodes [48]; Mg/Ca co-substitution synergistically stabilizes Ni/Mn-based P2 cathodes [49]; Li/Cu co-substitution simultaneously raises the intercalation voltage and improves cycling stability [52]; La doping enhances the cycling stability of O3 cathodes [53]; and Li/Mg/Si tri-doping (Na_0.7_Li_0.03_Mg_0.05_Ni_0.25_Mn_0.6_Si_0.07_O_2_) combines Li-driven layer expansion, Mg-driven Jahn–Teller suppression, and Si-driven oxygen-framework stabilization to deliver 165 mAh g^−1^ at 0.1 C with 90.5% retention over 200 cycles [54]. Na-layer pillars and TM-layer dopants play complementary roles (Figure 4c): the former restrain gliding and phase transitions, the latter harden the oxygen framework against dissolution and oxygen loss, and the most effective designs combine the two dopant classes and also differ in their primary effect on the cascade [48]. Na-layer pillars such as Ca^2+^ and Mg^2+^ act chiefly on the structural and mechanical links—restraining slab gliding and the associated volume change—whereas TM-layer dopants act chiefly on the chemical link by strengthening TM–O bonds against dissolution and oxygen release [41,48,51]. This is why co-doping that occupies both sites, or a pillar ion combined with a TM-layer stabilizer, generally outperforms a single-site dopant: it severs two links of the cascade simultaneously rather than one [48,49].

Closely related to the electrostatic pinning of the TMO_2_ slab is the challenge of surface alkalis, which act as chemical igniters of the failure cascade. While physical washing can remove them, it often damages the surface structure and leads to rapid re-contamination. A more sophisticated approach is the chemical conversion of NaOH/Na_2_CO_3_ into an inert, ionically conductive, and often mechanically compliant surface phase. For instance, Ca bulk doping with surface enrichment can convert residual alkalis into a protective Ca-rich surface phase and a rock-salt-like layer on the non-(003) facets [55]. This strategy simultaneously eliminates the harmful basic species and uses them as a resource to build a functional protective layer, shutting down the electrolyte oxidation/HF-generation cycle. Moreover, because the conversion layer is chemically bonded to the substrate, it provides a foundation for subsequent nanoscale engineering.

### 3.2. Interface Reconstruction and Intergrown Heterointerfaces (Nanoscale)

**Figure 5:**
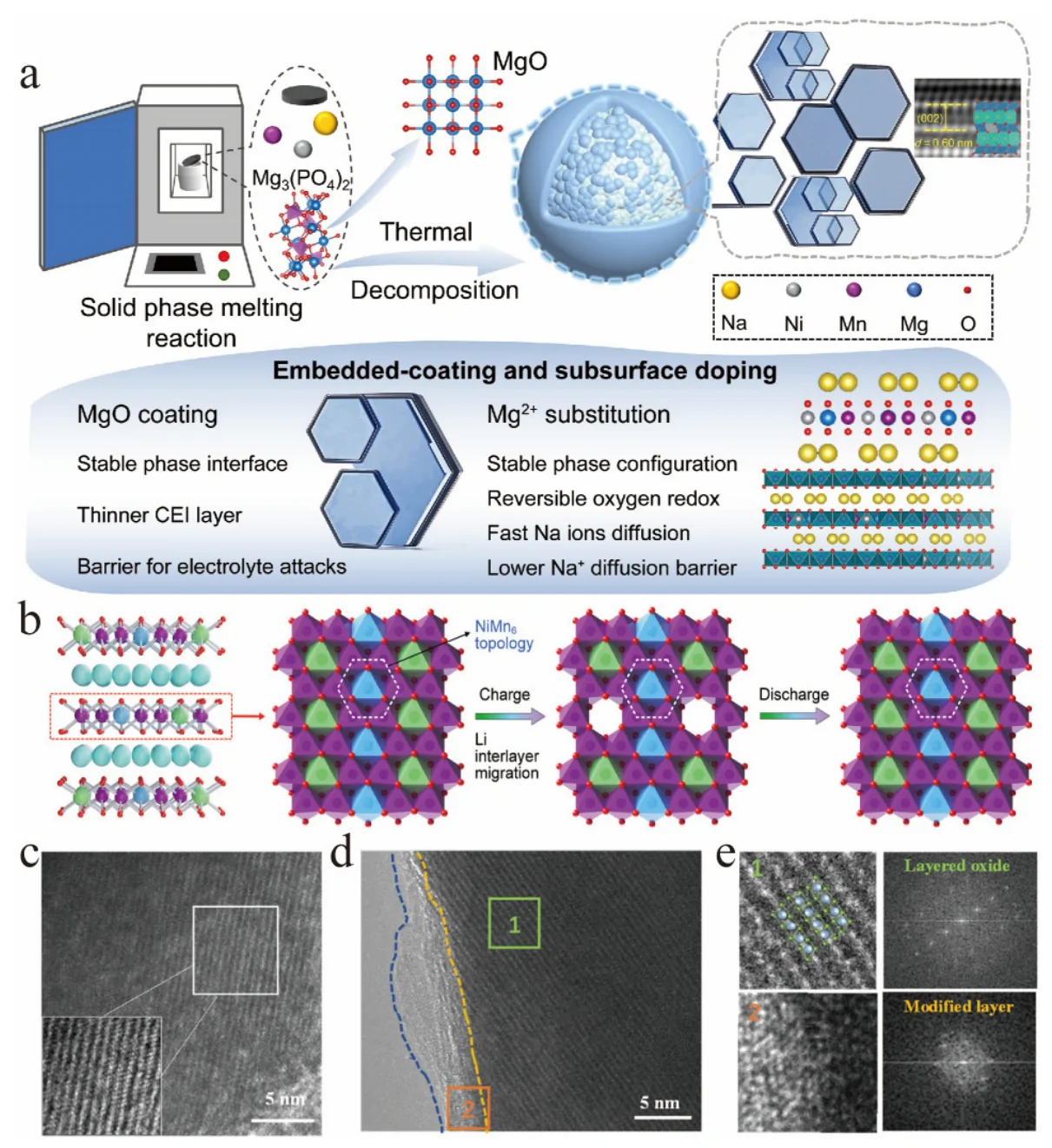
(**a**) Schematic diagram for in situ surface coating and subsurface gradient doping contrives P2-Na_0.67_Ni_0.33_Mn_0.67_O_2_ single crystal with a highly stable interface and structure [56]. (**b**) Superstructure evolution of Na*_x_*Ni_0.12_Li_0.18_Mn_0.7_O_2_ (NNLMO) upon charge and discharge [61]. (**c**) HRTEM image of Na_2/3_Mn_2/3_Cu_1/3_O_2_ (NMCO); (**d**) HRTEM image and (**e**) fast Fourier transform pattern of selected region 1 (green) and 2 (orange) [62]. Reproduced/Adapted with permission from the cited references.

Nanoscale modulation “stitches” the interface, interrupting the surface-reconstruction-to-cracking link. Coating-induced gradient doping is one route: during coating and annealing, coating elements diffuse into the near-surface lattice to form a chemically bonded “glue layer” that passivates the surface, homogenizes the interfacial Na^+^ concentration, and relieves stress concentration [41,56,57]. The NaCaPO_4_ coating with gradient Ca^2+^ doping, for example, pins the structure to uniformize phase transitions and suppress microcracks, delivering 105.3 mAh g^−1^ at 10 C and 81.0% retention over 300 cycles at 0.5 C, while remaining stable from −10 to 50 °C and up to 4.5 V [41]. The key to this design is the gradient itself: a uniform Ca concentration would stabilize the surface but leave a sharp doping boundary, whereas the gradient distributes the compositional and mechanical transition over a finite depth, homogenizing the Na^+^ distribution and the phase transformation within the particle [41]. An in situ 2–5 nm MgO coating plus a 3–5 nm Mg^2+^ subsurface gradient, derived from Mg_3_(PO_4_)_2_ decomposition (Figure 5a), gives P2-Na_0.67_Ni_0.33_Mn_0.67_O_2_ 100.3 mAh g^−1^ at 1 C and 80.8% retention over 500 cycles [56].

Epitaxial and conformal coatings with lattice matching are another route: by lowering the interfacial nucleation barrier, they achieve highly conformal coverage, which grows perpendicular to the (003) plane to form a lattice-anchored interface that couples ion conduction with air and chemical stability [41,58]. Atomic layer deposition (ALD) achieves Ångstrom-level thickness control through self-limiting reactions: ALD-grown ZnO/NiO/Al_2_O_3_ on Na_0.7_MnO_2_ (with 750 °C post-annealing) introduces near-surface doping confirmed by EELS [59], and a conformal ultrathin Al_2_O_3_ layer on O3-NaNi_1/3_Fe_1/3_Mn_1/3_O_2_ suppresses electrolyte decomposition and lowers interfacial resistance [60].

A third route exploits intergrown heterointerfaces for stress buffering and ion transport. O3/P2 and P2/P3 biphasic structures, as well as layered/spinel and layered/tunnel intergrowths, buffer lattice-mismatch stress at phase boundaries while the open frameworks of the spinel and tunnel phases provide fast-ion channels [26,40,63,64,65]. Lattice-coherent interfaces—where heterogeneous phases share atomic-scale lattice continuity—constrain interlayer sliding and phase degradation while improving ion kinetics and moisture stability [40]; the NiMn_6_ superlattice topology (Figure 5b) similarly enables high reversibility of both cationic and anionic redox [61]. Active-facet modulation and mechanochemical interface construction accelerate lattice-oxygen kinetics [66,67]. It should be stressed that the “spinel” phase here is a deliberately engineered benign intergrowth, fundamentally distinct from the rock-salt degradation product discussed in Section 2.2 [24,40]. Surface-reconstruction-induced Y-rich NaYO_2_ coatings [68] and Nb–Ti synergistic surface modification (Figure 5c–e) [62], by contrast, strengthen surface chemistry while inducing site-selective bulk substitution to stabilize the bulk, surface, and interface simultaneously. Lattice-coherent interfaces take several concrete forms: P2/O3 intergrowths raise capacity retention from roughly 40% to 68–81% over 200–500 cycles, P2/P3 biphasics contract the lattice at the shared TMO_2_ boundaries, and layered/tunnel intergrowths provide low-barrier ion pathways while the tunnel phase raises the surface contact angle (for example, from 102.2° to 118.9°) to improve moisture resistance [40]. The common ingredient in these nanoscale strategies is strong interfacial bonding rather than mere physical contact. A physically adsorbed coating can delaminate under the volume change in cycling, re-exposing the surface, whereas a lattice-coherent or chemically bonded layer moves with the lattice and continues to passivate [40,41]. This distinction explains why coating chemistry alone—without attention to the interface the coating forms—is an incomplete predictor of durability, and it is why the epitaxial and gradient approaches discussed above are often more effective than a nominally identical but mechanically detached layer [41,56].

### 3.3. Coating Thickness and Particle Architecture (Microscale)

**Figure 6:**
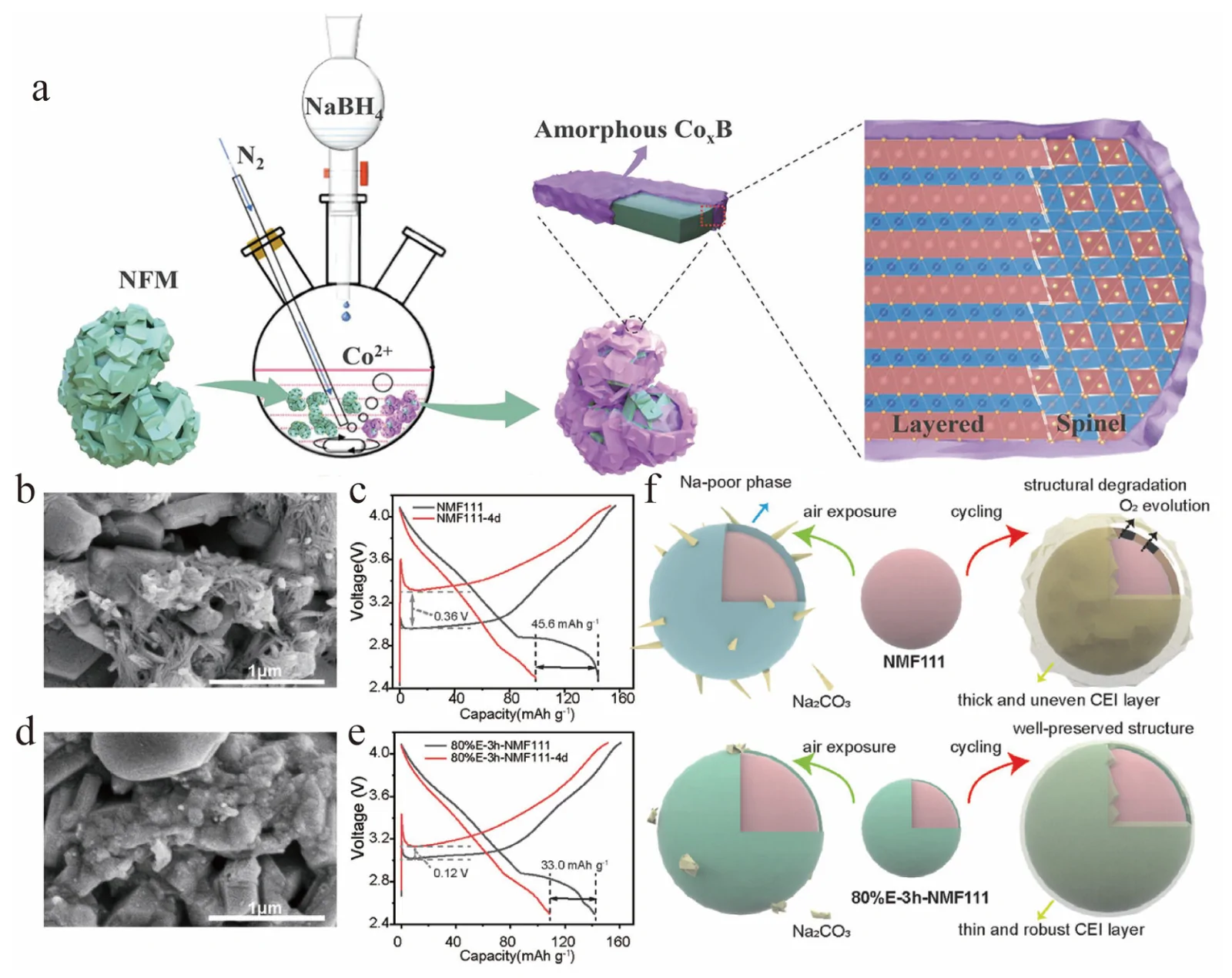
(**a**) Schematic diagram of the synthesis process for 1 wt%-Co_x_B@NaNi_1/3_Fe_1/3_Mn_1/3_O_2_. SEM images and the corresponding charge/discharge profiles of the aged [73]. (**b**,**c**) NaNi_1/3_Mn_1/3_Fe_1/3_O_2_ (NMF111) and (**d**,**e**) 80%E-3h-NMF111 in wet air (50% RH) for 4 days. (**f**) Schematic illustration of the surface evolution of NMF111 and 80%-3h-NMF111 in wet air or after long cycling [74]. Reproduced/Adapted with permission from the cited references.

Microscale modulation coordinates chemical shielding with global mechanical synergy at the single-particle level. Multifunctional coating thickness is the first consideration: metal-oxide and ceramic coatings (Al_2_O_3_, MgO, ZrO_2_, TiO_2_, NaNbO_3_, CeO_2_, Na_2_MoO_4_, among others) act simultaneously as physical barriers, HF scavengers, and residual-stress regulators, but their thickness has an optimum—too thin offers insufficient protection, and too thick blocks Na^+^ transport and sacrifices capacity [35,44,69,70,71,72]. A ~20–50 nm MgO coating, for instance, raises the 250-cycle retention of P2-Na_0.67_Mn_0.67_Ni_0.33_O_2_ from very low values to 70% and lifts the operating voltage to 3.66 V, with DEMS confirming suppressed oxygen evolution [44]. MgO coating combined with Mg^2+^ doping on Na_0.67_Mn_0.5_Fe_0.5_O_2_ suppresses TM dissolution and side reactions, reduces interfacial resistance, and alleviates the interlayer-spacing change, raising 1 C retention from 20% to 72% over 100 cycles [69]. Sn dual modification (8% Sn, bulk Sn^4+^ doping plus a surface Sn/Na/O rock-salt coating) doubles the 150-cycle retention of O3-NaNi_1/3_Fe_1/3_Mn_1/3_O_2_ in 2.0–4.1 V and delivers 81% retention over 200 cycles in full cells, with the Na^+^ diffusion coefficient after 100 cycles rising to 1.59 × 10^−12^ cm^2^ s^−1^ [35]. A 2–3% NaNbO_3_ coating markedly improves the 5 C rate and sub-0 °C cycling of P2-Na_2/3_Ni_1/3_Mn_2/3_O_2_; XRD shows that Nb does not enter the lattice (a physical rather than doping interaction), and XPS traces capacity to the Ni^4+/3+^ and Ni^3+/2+^ couples together with partially reversible oxide-to-peroxide oxidation [70]. Similarly, 2 wt% CeO_2_ raises the capacity and retention of P2-Na_0.67_Co_0.25_Mn_0.75_O_2_ to 135.93 mAh g^−1^ with 83.38% retention over 100 cycles, the improvement being attributed to suppressed Mn^3+^ dissolution and Jahn–Teller distortion together with slight Ce-induced lattice expansion [71]. In situ construction of a Co_x_B-spinel dual coating (Figure 6a) further stabilizes high-voltage cycling [73], and a Na-poor surface layer (Figure 6b–f) realizes structural, interfacial, and air stability in a single “three-in-one” step [74]. The existence of an optimal thickness has a second, less obvious origin: beyond blocking Na^+^ transport, an over-thick inactive coating can consume Na^+^ to form secondary phases and can itself become a site of interfacial resistance [16,44]. The optimum is therefore set not only by the balance between shielding and transport but also by the chemical reactivity of the coating with the cathode surface. This is why the field has increasingly moved toward thin, ionically conductive, or in situ-generated layers, which sidestep the trade-off altogether [58,59,75].

**Figure 7:**
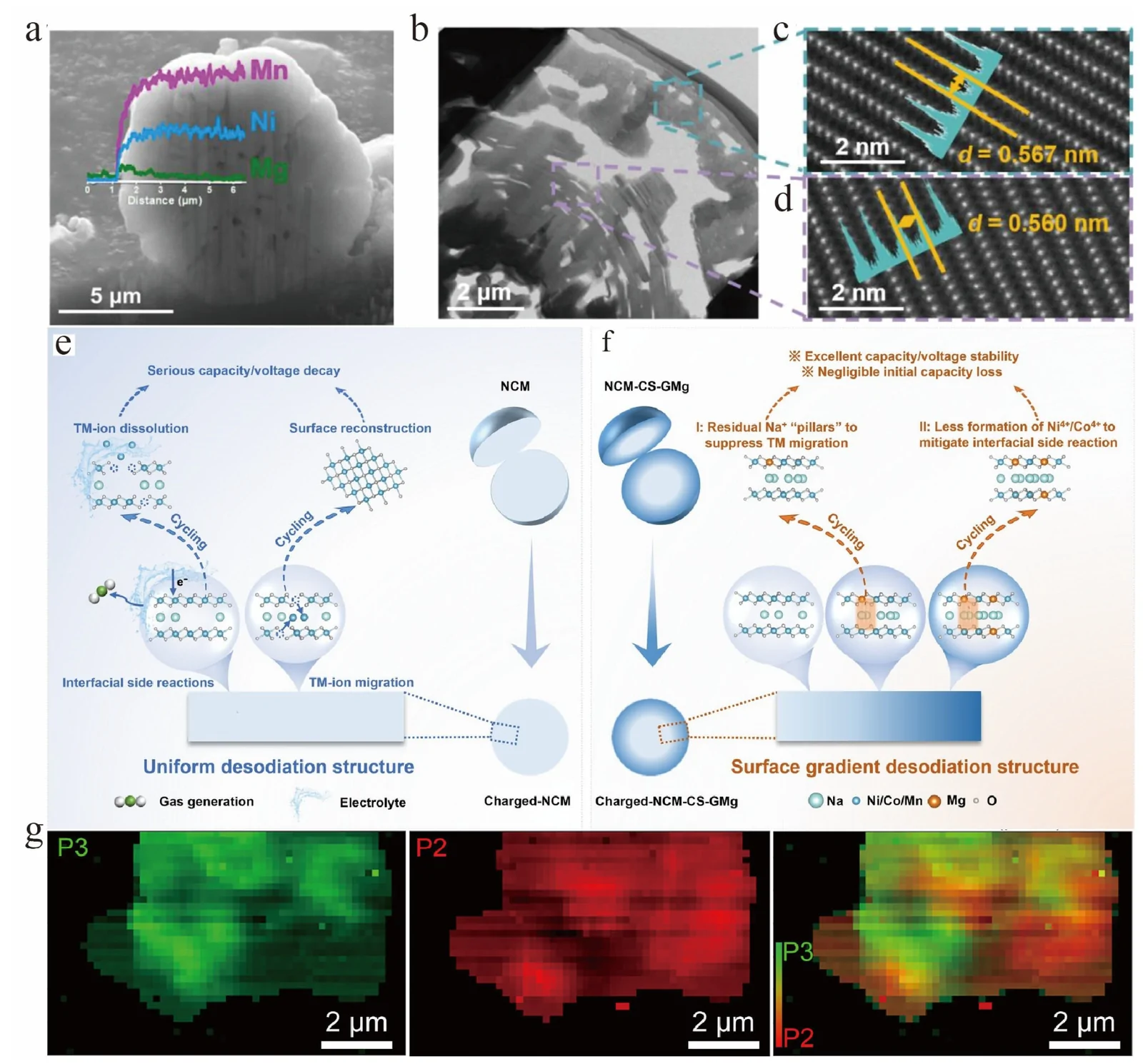
(**a**) Cross-sectional SEM image of the NM–Mg–CS and the corresponding elemental line scan from shell to core. (**b**) HAADF-STEM image of a slice of the NM–Mg–CS particle containing the core and the shell. (**c**,**d**) HAADF-STEM images of the blue–green rectangle and violet rectangle in (**b**) [77]. (**e**) Schematic illustration of the degradation mechanism in NCM. (**f**) Surface gradient desodiation structural design concept for Na_0.72_Ni_0.20_Co_0.21_Mn_0.55_Mg_0.036_O_2_ (NCMCS-GMg) [78]. (**g**) Diffraction mappings of Na_0.8_(Ni_0.33_Mn_0.67_)_0.8_Li_0.1_Ti_0.1_O_2_ (NMLTO) particles using XRF-XRD [79]. Reproduced/Adapted with permission from the cited references.

Core–shell and concentration-gradient architectures form the second lever: core–shell structures (such as an O3 core with a P2 shell) and concentration-gradient designs extend protection from the surface to the whole particle, achieving surface stability together with bulk mechanical synergy, while single-crystallization removes grain boundaries and crack sources altogether [40,45,76]. A P2-Na_0.67_[Ni_0.25_Mn_0.75_]O_2_ core enclosed by a Mg-substituted P2-Na_0.67_[Ni_0.21_Mn_0.71_Mg_0.08_]O_2_ shell (Figure 7a–d), for example, suppresses the P2→O2 transition through a confinement effect and retains 81% capacity over 1000 cycles at 5 C [77]. A Mg-gradient shell (Na_0.72_Ni_0.20_Co_0.21_Mn_0.55_Mg_0.036_O_2_) induces a surface gradient desodiation (Figure 7e,f) that inhibits TM migration and the layered-to-rock-salt transition, sustaining 3000 cycles with only 0.127 mV cycle^−1^ voltage decay [78], and a coherent gradient from a rocksalt surface to a P2/P3 bulk heterostructure (Figure 7g) delivers 94 mAh g^−1^ at 5 C with 76% retention over 1000 cycles [79]. Gradient structural design that “locks” interfacial oxygen provides yet another route to air-stable, high-performance layered oxides [57]. Ti substitution, for example, promotes the growth of ~10 μm single crystals that suppress multiphase transitions and irreversible anion redox while markedly improving air stability [45]. Between core–shell and concentration-gradient designs, the gradient is generally more robust because it removes the sharp composition boundary where lattice mismatch and mechanical stress concentrate; a sharp shell–core interface can delaminate or crack, whereas a smooth gradient distributes the strain over a wider region [76,78]. Core–shell structures nevertheless remain attractive when the shell needs to be compositionally distinct—for example, a stabilizing Mg-rich or tunnel-phase shell over a high-capacity core—and the two concepts are increasingly blended into single particles with both a compositional shell and a graded transition [77,79]. Single-crystallization removes grain boundaries entirely and is therefore the most direct route to mechanical robustness, but it trades this for slower solid-state diffusion and, in some cases, a higher sensitivity to surface reconstruction because a single large crystal exposes fewer, but larger, (003) facets [45]. The choice among polycrystalline gradient, core–shell, and single-crystal morphologies is thus a genuine design trade-off rather than a strict hierarchy.

## 4. From Single-Axis Modulation to Synergistic and System-Level Design

Because the cascade is coupled, a strategy that severs only one link leaves the loop running elsewhere. This chapter therefore turns from individual strategies to their combination and to the second, electrolyte-side interface. It makes two arguments. First, the most effective designs pair a surface (kinetic) function with a bulk (thermodynamic) function, and a chemical function with a mechanical function—not as an accumulation of tricks but as a deliberate division of labor. Second, interface engineering is incomplete if it stops at the particle: the cathode–electrolyte interphase, and the cross-electrode interface beyond it, are equally part of the cascade. Throughout, “active regulation” is used as a spectrum rather than a binary label: residual-alkali conversion is its purest form, while the coating-as-dopant-reservoir and the anion-derived CEI are intermediate forms, in which the interface is co-produced with the electrochemical and thermal history of the cell [47,80,81]. Table 1 collects representative recent examples of each modulation strategy, together with the specific material and the electrochemical outcome. The examples are chosen to illustrate, with concrete data, the effect that each strategy class achieves; because test conditions vary across studies, the numbers should be compared within a strategy class rather than across the whole table.

### 4.1. The Coating-Plus-Doping Cascade

The strategy that most directly embodies this division of labor is the coating-plus-doping cascade anticipated in Section 3.1: the coating is chosen not merely as a barrier but as a dopant reservoir that, during annealing, releases its cations into the near-surface lattice while consuming residual alkali [47,88]. Three features distinguish this from coating or doping used alone. First, the dopant gradient is self-aligned with the failure gradient—it peaks at the surface, where degradation is most severe, and decays inward [41,89]. Second, the coating and the dopant divide the work: the coating handles chemical shielding and ion conduction, while the gradient doping uniformizes the phase transformation and relieves lattice mismatch [41,56,87]. Third, because the coating is formed from the cathode’s own residual alkali, the interface is “active” in the sense defined above [47,81]. Representative realizations—NaCaPO_4_/Ca^2+^ [41], CaZrO_3_/Ca/Zr/F [38], Na_3−2x_Zn_x_PO_4_/Zn^2+^ [89], CaTiO_3_/Ca/Ti [82], and Li/Ti/Co with AlPO_4_ [87]—were introduced with their quantitative outcomes in the above sections; what matters here is the shared logic rather than the individual numbers.

### 4.2. Mechano-Electrochemical Synergistic Interfaces

**Figure 8:**
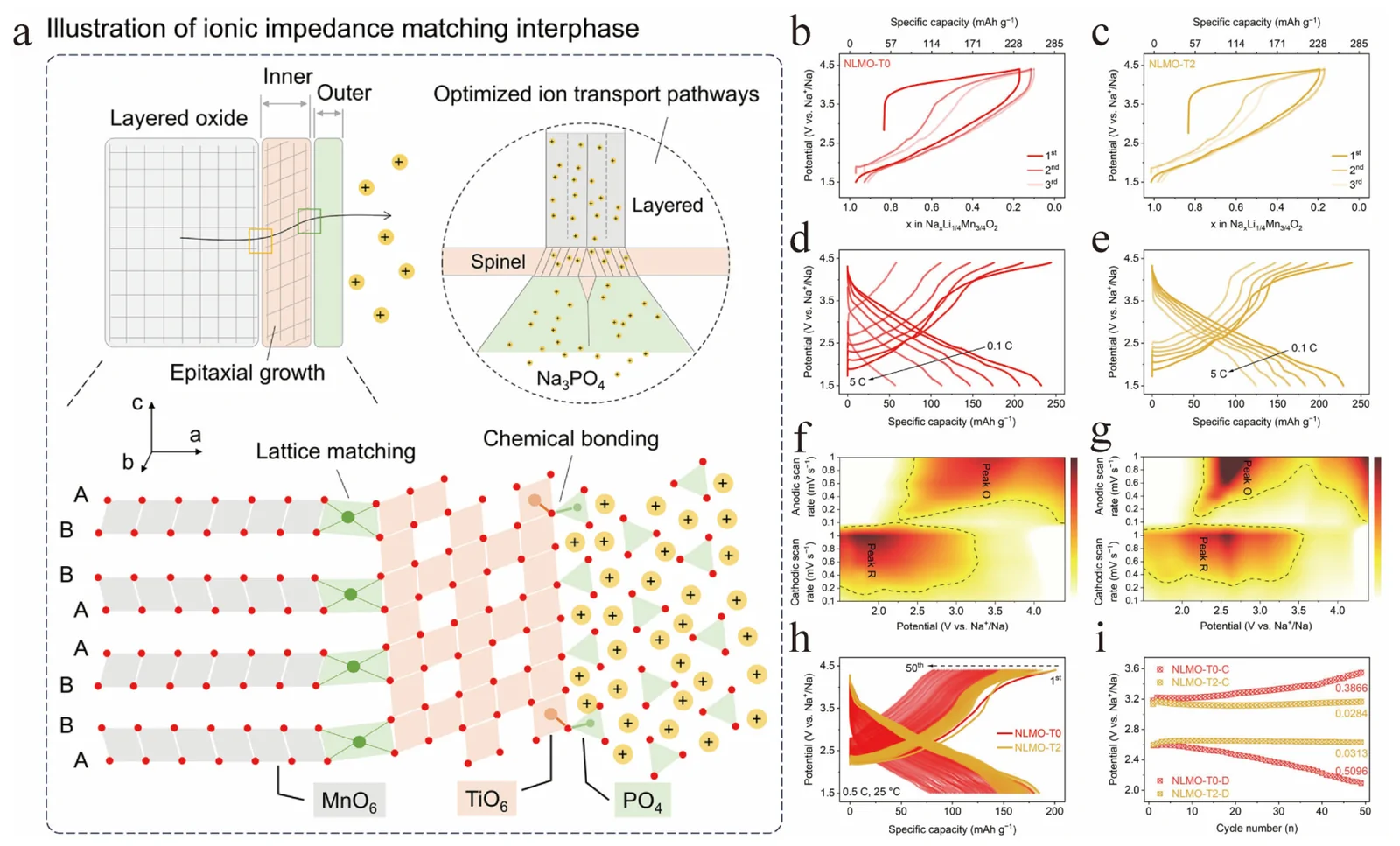
(**a**) Proposed ionic impedance matching interphase: a conformal Na_3_PO_4_ shell and a Ti-rich spinel-like underlayer form a coherent interface with the layered bulk, enabling continuous Na^+^ transport while alleviating lattice and impedance mismatches. The first three charge–discharge cycles of (**b**) NLMO-T0 and (**c**) NLMO-T2 at 0.1 C within 1.5–4.4 V. Charge–discharge profiles of (**d**) NLMO-T0 and (**e**) NLMO-T2 at different rates. CV contour plots of (**f**) NLMO-T0 and (**g**) NLMO-T2 recorded at various scan rates. (**h**) Comparison of long-term cycling stability at 0.5 C. (**i**) Comparison of voltage decay over cycling [75]. Reproduced/Adapted with permission from the cited references.

Mechano-electrochemical synergy is key to countering chemo-mechanical cracking. Flexible organic and inorganic hybrid coatings accommodate volume strain better than rigid oxides, and some even show crack self-healing. A self-assembled DHP (C_32_H_67_O_4_P) monolayer (~6 nm), for example, bonds to surface hydroxyl groups to form a hydrophobic layer that gives NFM@DHP 87.3% retention over 330 cycles at 1 C (versus 54.5% for the unmodified material) and retains 118 mAh g^−1^ after seven days in humid air (30 °C, 50% relative humidity) [84]. Ion-conducting coatings simultaneously passivate chemically and lower interfacial transport resistance: NASICON-type NaTi_2_(PO_4_)_3_, Na_2_Ti_3_O_7_, and Na_3_PO_4_ fast-ion conductors block electrolyte attack while providing low-barrier channels for Na^+^ [58,75,83]. A thermally converted NaTi_2_(PO_4_)_3_ interphase with an Na_3_PO_4_ outer layer and a Ti-rich spinel-like inner layer realizes an ionic-impedance-matched interface (Figure 8a) with ~230 mAh g^−1^ and <0.05 V voltage decay (Figure 8b–i) [51] [75]. A 5–8 nm Na_2_Ti_3_O_7_ coating converted from surface residual alkali gives O3-Na_0.95_Ni_1/3_Fe_1/3_Mn_1/3_O_2_ 101.9 mAh g^−1^ at 10 C and 85.66% retention over 200 cycles at 1 C; its uniform coverage reduces the electrolyte contact angle from 108° to 79° [58]. The contrast between the two classes is instructive: rigid oxides such as MgO and Al_2_O_3_ excel at chemical shielding but impede Na^+^ transport if too thick, whereas ion-conducting and flexible layers reconcile passivation with transport and strain accommodation [44,58,75,83,84]. The mechano-electrochemical frontier also reveals a subtlety about flexibility: a coating need not be soft to accommodate strain if it is lattice-matched and moves with the host, and conversely an overly compliant organic layer may passivate but offer little mechanical reinforcement [75,84]. The most robust designs therefore combine an outer flexible or ion-conducting phase for passivation and transport with an inner, lattice-anchored phase for mechanical coupling, as in the Na_3_PO_4_/spinel bilayer discussed above [75].

### 4.3. Thermodynamic and Kinetic Co-Stabilization Under Extreme Conditions

**Figure 9:**
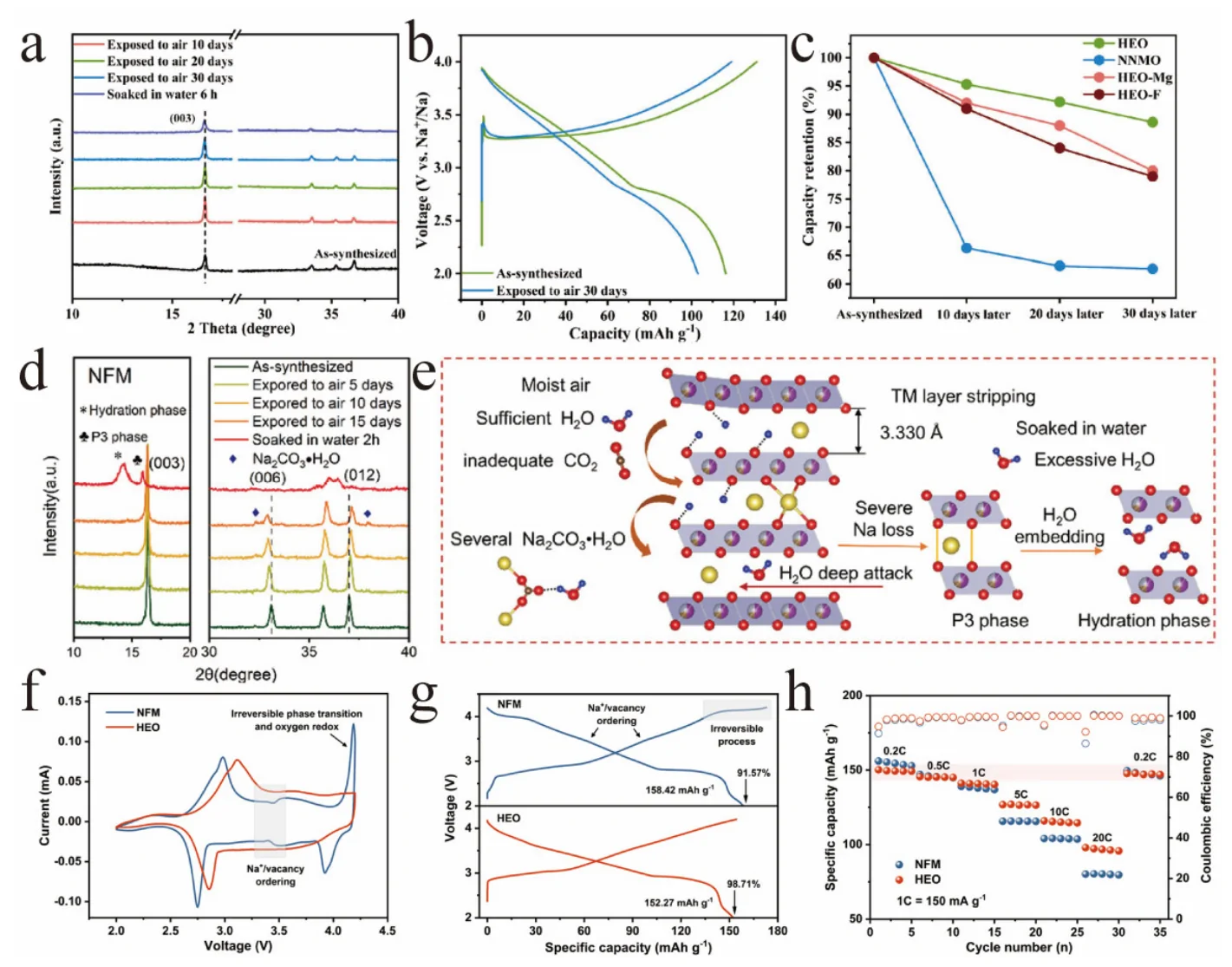
(**a**) The XRD patterns of HEO after different exposure times to air and after soaking in water. (**b**) The initial charge–discharge curves of as-synthesized HEO and HEO exposed to air for 30 days at 2 C. (**c**) The capacity retention of HEO, NNMO, HEO-F, and HEO-Mg varied with the exposure time in air [86]. (**d**) The XRD patterns of NFM after different exposure times to air and after soaking in water. (**e**) The mechanism demonstrations of NFM exposed to air and soaked in water [39]. (**f**) CV curves of NFM and HEO at 0.1 mV s^−1^ for the first cycle. (**g**) Initial galvanostatic charge/discharge curves of NFM and HEO at 0.1 C (1 C = 150 mA g^−1^). (**h**) Rate performance of NFM and HEO [98]. Reproduced/Adapted with permission from the cited references.

Deep desodiation places still higher demands on the interface. In the aggressive environment of high potential, the interface must balance “sacrificial” consumption of reactive species against long-term stable passivation. A buffered fluorination strategy using Na_3_FeF_6_, which releases F^−^ at a constant rate and constant acidity, builds an armor-like interface on O3-Na(NiFeMn)_1/3_O_2_ in humid conditions; the surface fluorination weakens the ligand-field splitting of Fe and suppresses Fe dissolution and the O3′ phase, retaining 84.7% over 1000 cycles in 2.0–4.0 V [85]. High-entropy and high-entropy-plus-coating interfaces provide a second approach: multi-principal-element TM disorder retards surface reconstruction and elemental segregation, thermodynamically suppressing phase transitions and air attack [90,91,92,93,94,95,96,97,98,99]. The “high-entropy lattice-pinning” cathode (O3-Na_0.96_Ca_0.02_Ni_0.27_Fe_0.2_Mn_0.35_Al_0.02_Cu_0.1_Ti_0.05_O_2_) anchors the Na layer with Ca^2+^ to achieve a 1.1% volume strain, 80.1% retention over 1000 cycles at 5 C, and 264.87 Wh kg^−1^ in full cells [43]. A cation–anion co-doped high-entropy O3 cathode (NaNi_0.3_Mn_0.3_Fe_0.2_Ti_0.1_Cu_0.06_Mg_0.04_O_1.95_F_0.05_) retains 88.6% of its initial capacity after 30 days of air exposure (Figure 9a–c) [86], and the NCFMMT high-entropy cathode sustains 86.8% retention over 500 cycles at 5 C with excellent air stability [100]. High-entropy design also extends to solid-state cells and to Co-free oxyfluorides, broadening the practical reach of the strategy [101,102]. A high-entropy O3-Na_0.85_Li_0.1_Al_0.02_Sn_0.08_Cu_0.1_Ti_0.1_Ni_0.3_Mn_0.3_O_2_ cathode, for instance, stabilizes the interlayer oxygen charge and a reversible P3–OP2–P3 transition (Figure 9d,e), retaining 96.12% of its capacity after five days in air and cycling 900 times over 2.0–4.3 V [39]; the same approach delivers 141 mAh g^−1^ at 0.2 C, 85 mAh g^−1^ at 20 C, and 96% retention over 400 cycles in a polymer-electrolyte solid-state cell [101]. Compositional descriptors further sharpen the design: a low-R high-entropy O3 oxide (R = d(O–Na–O)/d(O–TM–O) = 1.288) contracts the Na layer to suppress active-sodium loss, delivering 142.83 mAh g^−1^ at 0.2 C while retaining its structure after 24 h at 50% relative humidity [103], and cation–anion dual-site high-entropy doping (NaNi_0.25_Fe_0.14_Mn_0.3_Li_0.1_Ti_0.15_Cu_0.03_Zn_0.03_O_1.94_F_0.06_) strengthens TM–O covalency to deliver 152.27 mAh g^−1^ with 73.69% retention over 500 cycles (Figure 9f–h) [98]. This family illustrates a division of labor within the wide-temperature/high-voltage frontier: high-entropy composition provides thermodynamic bulk stability against phase transitions and air attack, while surface coatings provide kinetic interfacial passivation, and the two are increasingly combined because neither alone covers both fronts [39,43,86,90]. The distinction between thermodynamic and kinetic stabilization is worth making precise. High-entropy composition raises the free-energy barrier to phase transitions and lowers the driving force for elemental segregation, but it does not by itself shield the surface from electrolyte attack; conversely, a coating shields the surface kinetically but does not stabilize the bulk lattice against the phase transitions that continue beneath it [43,86]. The two are therefore not substitutes but partners, and the strongest recent results arise from their combination, which stabilizes both the reaction the cathode undergoes and the interface through which it must pass [39,43,90].

### 4.4. CEI Engineering: Co-Design of the Second Interface

**Figure 10:**
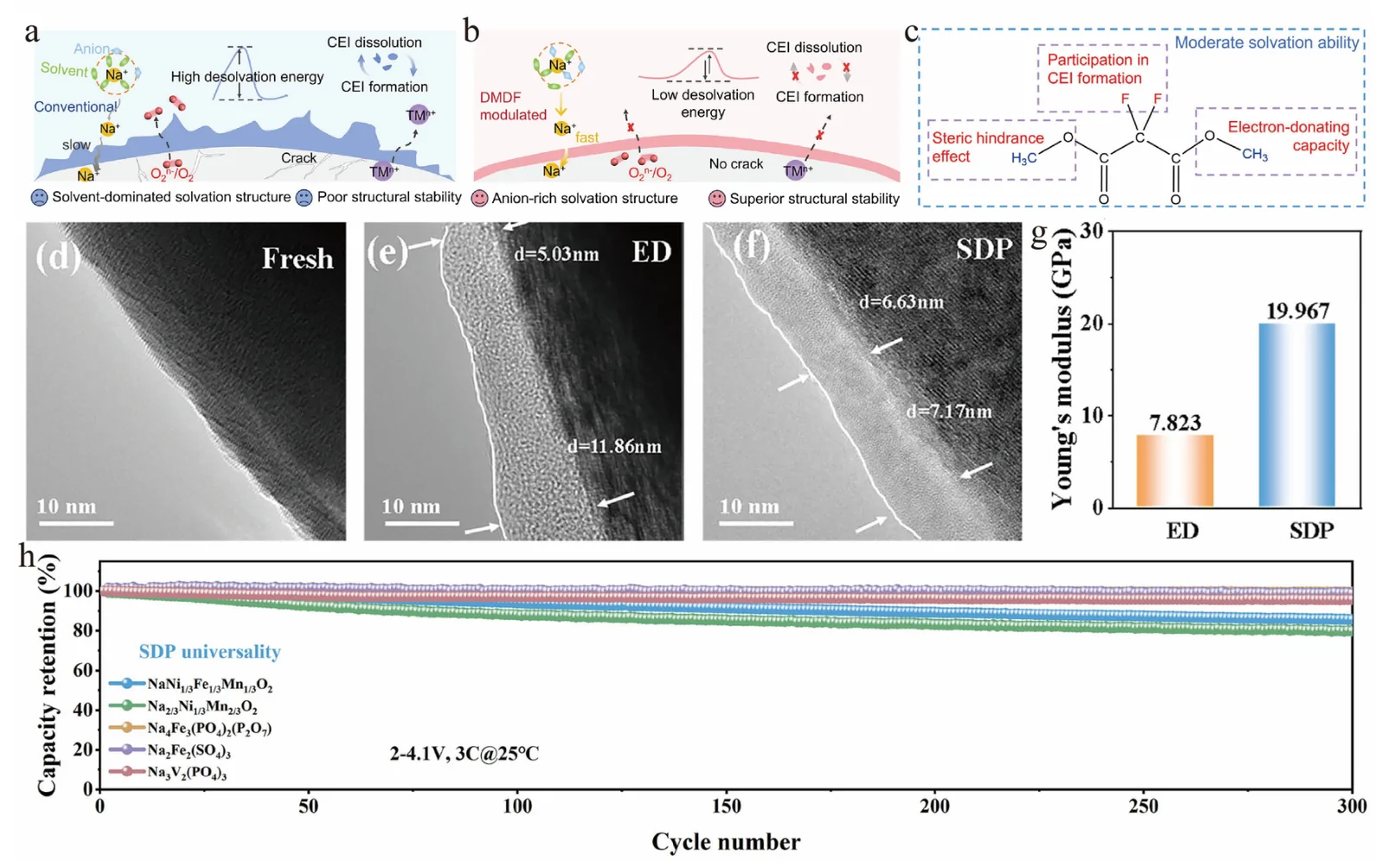
Schematic illustration of interfacial chemistry on (**a**) conventional carbonate-based electrolyte and (**b**) designed electrolyte. (**c**) Molecular structures of DMDF [81]. (**d**–**f**) TEM Images of the fresh NaNFM cathode and the NaNFM cathodes cycled in ED and SDP electrolyte after 300 cycles within the range of 2–4.1 V at 3 C. (**g**) The average Young’s modulus of cycled NFM electrodes after 300 cycles within the range of 2–4.1 V at 3 C. (**h**) SDP universality: cycling performance of different cathode materials (O3, P2, NFPP, NFS, and NVP) within the voltage range of 2–4.1 V at 3 C [80]. Reproduced/Adapted with permission from the cited references.

**Figure 11:**
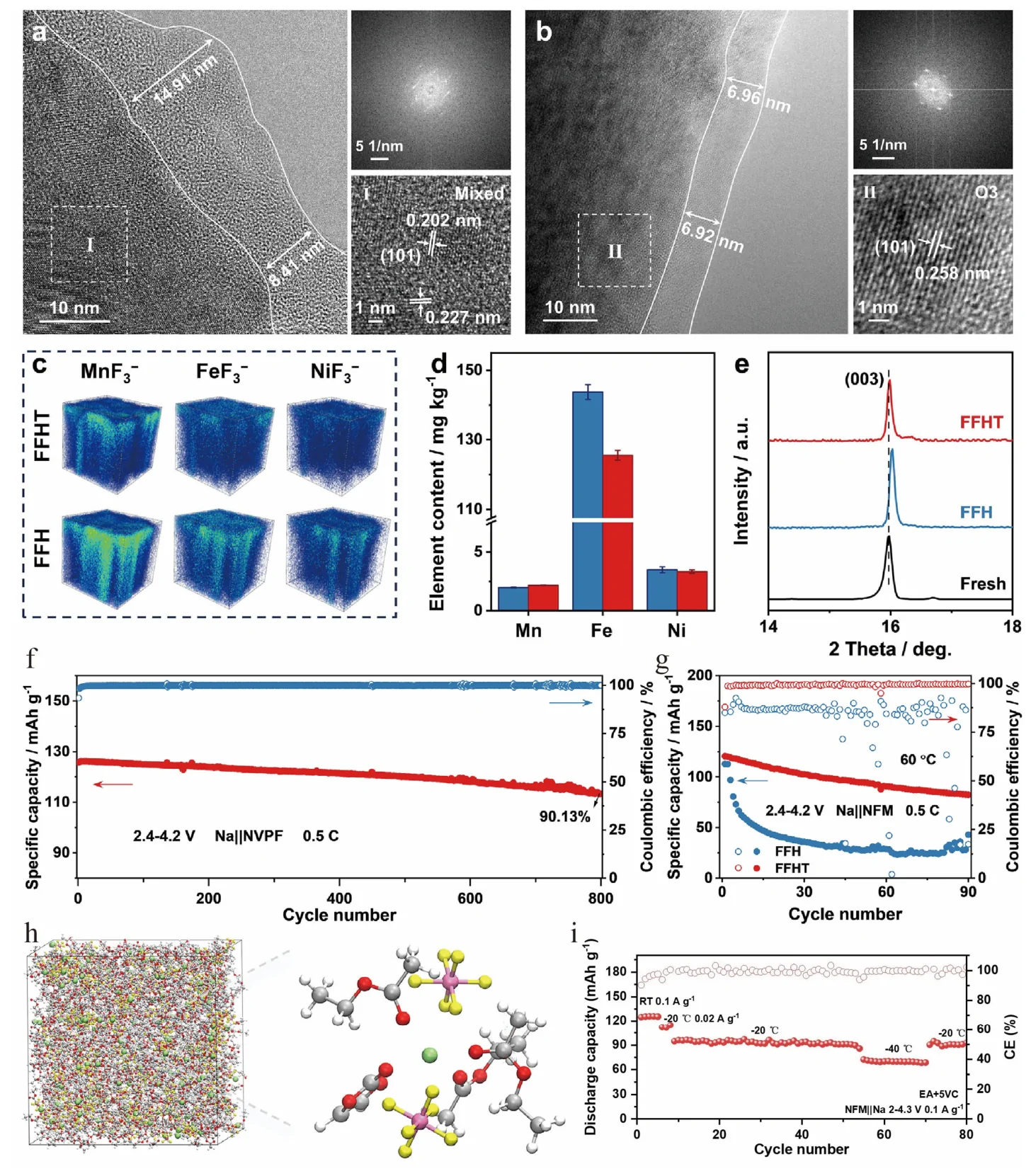
Characterization of the structural degradation and interfacial uniformity of NFM cathodes. HR-TEM images and their corresponding area electron diffraction patterns and magnified images of the cycled NFM cathode surface with (**a**) FFH and (**b**) FFHT electrolytes; (**c**) depth profiles of the TOF-SIMS intensity of MnF3-, FeF3- and NiF3- fragments in the CEI film formed in FFH and FFHT electrolytes; (**d**) the Mn, Fe and Ni content on the sodium anode and separator of the Na||NFM batteries with different electrolytes after 50 cycles was detected by ICP-OES; (**e**) XRD patterns for fresh NFM cathodes and NFM cathodes after 50 cycles with different electrolytes. (**f**) Cycling performance of the Na||NVPF battery at 0.5 C between 2.4 and 4.2 V and (**g**) high-temperature performance of the NFM cathode at 60 °C [105]. (**h**) Snapshots of the solvation structure of EA + 5VC. (**i**) Cycling performance of NFM||Na cells using EA + 5VC in the voltage range of 2–4.3 V at 0.1 A g^–1^ under −20 °C and −40 °C conditions at 0.1 A g^–1^ [110]. Reproduced/Adapted with permission from the cited references.

Cathode-interface stability depends not only on the material itself but also on the coupling between the electrolyte and the cathode’s surface catalytic activity. The first lever coordinates solvation structure with surface chemistry: co-solvents, additives, or locally high-concentration designs draw anions into the solvation sheath so that they decompose preferentially into an inorganic-rich (NaF-dominated), high-modulus, dense CEI [80,81,104,105,106,107,108,109]. The dimethyl difluoromalonate (DMDF) co-solvent builds a NaF-rich CEI (Figure 10a–c) that sustains 80% retention over 600 cycles in 1.5–4.5 V and 304.5 Wh kg^−1^ in full cells [81]. An SL/DMC/PFPN ternary-solvent electrolyte forms an anion-rich CEI (modulus 19.97 GPa, ~7 nm thick, Figure 10d–g) with 85.72% retention over 300 cycles at 3 C (Figure 10h) [80]. The DFEC additive forms a fluorine-rich CEI that keeps NFM at 78.36% over 200 cycles at 4.2 V with an initial Coulombic efficiency of 93.74% [106]. TMSI-induced anion interphases raise NFM retention at 55 °C from 51.5% to 74.4% [109], and a tris(trimethylsiloxy)borate additive both stabilizes the CEI and promotes Na^+^ desolvation [107]. A “bulletproof” CEI, outer-strong and inner-flexible (Figure 11a–e) through additive-guided solvent coordination, stabilizes operation at 60 °C and high voltage (Figure 11f,g) [105].

The second lever optimizes the cathode and anode interfaces jointly: electrolyte engineering stabilizes the cathode CEI and the anode SEI simultaneously, suppressing the cross-electrode “crosstalk” in which dissolved TMs migrate to and degrade the anode SEI [104,110,111,112,113]. A VC-modified ethyl-acetate electrolyte (Figure 11h), for instance, builds a gradient CEI with a fluorine-rich organic exterior and a NaF/Na_3_PO_4_ inorganic interior that enables 4.5 V operation down to −40 °C (Figure 11i) [110]; non-solvating fluorinated-ether additives decouple cathode and anode stabilization to reach 99.92% anode efficiency and 1200-cycle cathode stability [111]. These advances build on the early recognition that a robust hard-carbon SEI is indispensable for full-cell operation [4], and together they integrate the cathode and anode interfaces at the cell level [104,108,112,113]. A recurring theme is that the CEI must be simultaneously passivating, mechanically robust, and ionically conductive: NaF-rich inorganic layers provide high modulus and electronic insulation, while organic outer layers accommodate strain, so the optimum is a gradient, outer-flexible/inner-strong architecture rather than a homogeneous film [80,81,105,110]. A parallel design axis concerns the origin of the CEI. Solvent-derived interphases tend to be organic-rich and mechanically soft, whereas anion-derived interphases—formed when anions enter the first solvation shell and decompose preferentially—are inorganic-rich, high-modulus, and electronically insulating [80,104,111]. Steering the CEI toward the anion-derived regime, through high-concentration or non-solvating designs, is thus a general route to the outer-flexible/inner-strong architecture described above, and it explains why seemingly minor electrolyte formulation changes can produce large differences in cathode lifetime [80,108,111].

The benefits of each strategy carry measurable costs. Inactive coatings add mass and can raise interfacial resistance once thick; ion-conducting coatings improve transport but often require multi-step or in situ synthesis; high-entropy compositions improve bulk stability but dilute redox-active capacity and raise material cost; gradient and core–shell particles demand complex precipitation control; and single crystals trade mechanical robustness for slower solid-state diffusion. Coating uniformity, dopant cost, synthesis complexity, and reproducibility—rarely reported—are therefore the practical bottlenecks. Table 2 consolidates this cascade-to-strategy correspondence: for each link of the feedback network, it lists the dominant length scale, the modulation strategies that interrupt that link, and representative examples discussed in Section 3 and Section 4. Together with the strategy-by-strategy summary of Table 1, it provides a roadmap from failure mechanism to intervention.

## 5. Summary and Outlook

Synthesizing the failure mechanisms and modulation strategies above, we distill five actionable principles. First, target the weakest link: modulation should sever the most vulnerable link of the cascade rather than cover everything, stabilizing lattice oxygen first in oxygen-releasing high-voltage systems and converting residual alkali or hydrophobically passivating the surface first in air-sensitive systems. Second, do not neglect chemo-mechanical balance: chemical passivation and mechanical buffering must be assigned according to the spatial origin of failure, with coatings handling chemical shielding and doping or gradients handling mechanical stabilization. Third, turn liabilities into assets: convert “failure precursors” such as residual alkali and phase transitions into coating precursors or interfacial pinning resources, shifting from passive removal to active utilization. Fourth, match the scale: suppress gliding at the atomic scale, stitch interfaces at the nanoscale, and coordinate global mechanics at the microscale so that each intervention matches the length scale of the failure it addresses. Fifth, integrate at the system level: interface engineering must extend beyond the cathode alone to the co-design of the CEI and the anode interface. Three recurring pitfalls illustrate these principles in reverse: overly thick inactive coatings improve passivation at the cost of transport; doping without a chemically stable interface can leave the reconstructed surface unprotected; and optimizing the cathode in isolation ignores the cross-electrode crosstalk that ultimately limits full-cell life. Operationally, the five principles can be collapsed into a single question to be asked of any candidate interface: which link of the cascade does this design sever, and does its length scale and chemical character match that link?

Three directions merit attention. Responsive interfaces—self-healing, environment-adaptive dynamic interfaces—remain a challenging frontier [46]. AI-accelerated rational design is a second opportunity: as the composition–structure–property relationships of high-entropy and multi-component systems grow more complex, machine learning and descriptors such as the electronegativity-entropy weight may enable high-throughput screening of interface compositions [91,101,114]. Finally, integrated validation under extreme conditions—wide-temperature, high-voltage, high-loading pouch, and full cells—will be the ultimate yardstick for any interface strategy [26,81,115]. Progress on these three fronts is mutually reinforcing: better characterization and modeling supply the data that machine-learning descriptors require, and robust interfaces validated in realistic cells are what ultimately convert mechanistic insight into a manufacturable cathode. Operationally, the five principles translate to two core practices. First, identifying the initiating link by combining operando XRD (phase transition), DEMS (gas evolution), and post-mortem STEM/EELS (surface reconstruction) within a single cell: the technique that first detects an irreversible signature can be used to pinpoint the trigger. Second, standardizing baseline test conditions to make wide-temperature, high-voltage, and long-life performance claims comparable: all such claims should be reported with consistent experimental parameters, including a conventional electrolyte, a hard-carbon anode, a defined voltage window, C-rate, operating temperature, and cathode loading. If there is a single message of this review, it is that interfacial failure in layered sodium cathodes is best understood—and best treated—as a coupled, multi-scale phenomenon rather than a list of independent defects. The strategies that have moved the field forward are those that respect this coupling, either by severing the cascade at its weakest link or by turning its own precursors into protective phases. We expect the next generation of interfaces to push this logic further, from static protection toward interfaces that actively sense and respond to the state of the electrode.

## Figures and Tables

**Table 1:** Representative surface/interface modulation strategies for layered oxide cathodes and their electrochemical outcomes.

Strategy	Material	Targeted Failure Mechanism	Key Electrochemical Performance	Main Limitation/Trade-Off	Ref
Cu/Ca dual-site co-doping	O3 layered oxide	Slab gliding/phase transition	86.02 mAh g^−1^ at 10 C (2.4 A g^−1^, 2.0–4.0 V), 80.64% retention after 300 cycles.	Pillar ion may occupy Na sites	[48]
Li/Mg/Si tri-doping	P2-Na_0.7_Li_0.03_Mg_0.05_ Ni_0.25_Mn_0.6_Si_0.07_O_2_	Jahn–Teller/oxygen framework	165 mAh g^−1^ at 0.1 C; 90.5% retention over 200 cycles	Multi-dopant synthesis control	[54]
Residual-alkali conversion (phosphate-type)	Na layered oxides	Air instability/surface initiator	184.93 mAh g^−1^ at 1 C (160 mA g^−1^, 2.0–4.3 V), coulombic efficiency: 91.43%.	Requires residual alkali present	[47]
NaCaPO_4_ coating + gradient Ca^2+^ doping	O3 layered oxide	Microcrack/phase transition + air	105.3 mAh g^−1^ at 10 C; 81.0% over 300 cycles at 0.5 C; 82.9% (pouch, 300 cycles)	Epitaxial/gradient control	[41]
CaZrO_3_ perovskite + Ca/Zr/F co-doping	O3-NaNi_1/3_Fe_1/3_Mn_1/3_O_2_	Fe^4+^ Jahn–Teller/OP2 phase	83.8% retention over 300 cycles at 2 C	Multi-element, cost	[38]
CaTiO_3_ dielectric + Ca/Ti co-doping	P2-Na_2/3_Ni_1/3_Mn_2/3_O_2_	Oxygen release	81.2 mAh g^−1^ at 10 C (1.5–4.5 V)	Dielectric layer adds mass	[82]
MgO coating	P′2-Na_0.67_Mn_0.67_Ni_0.33_O_2_	HF corrosion/TM dissolution	>90 mAh g^−1^ at 50 mA g^−1^; 70% retention over 250 cycles	Insulating; thickness-sensitive	[44]
Sn dual modification (bulk Sn^4+^ + surface Sn/Na/O)	O3-NaNi_1/3_Fe_1/3_Mn_1/3_O_2_	Phase transition + surface	Doubled retention over 150 cycles; 81% over 200 cycles (full cell)	Sn cost	[35]
NaNbO_3_ coating	P2-Na_2/3_Ni_1/3_Mn_2/3_O_2_	Surface/oxygen redox	46.4 mAh g^−1^ at 5 C; sub-0 °C cycling	Physical (non-bonding) layer	[70]
CeO_2_ modification	P2-Na_0.67_Co_0.25_Mn_0.75_O_2_	Mn3+ dissolution/Jahn–Teller	135.93 mAh g^−1^; 83.38% retention over 100 cycles	Slight Ce incorporation	[71]
ALD oxide (ZnO/NiO/Al_2_O_3_)	P2-Na_0.7_MnO_2_	CEI/surface	ZnO: 190 mAh g^−1^; Al_2_O_3_: 178 mAh g^−1^ at 0.2 C (1 C = 200 mA g^−1^, 1.8–4.0 V)	ALD cost/scalability	[59]
NASICON NaTi_2_(PO_4_)_3_ coating	O3-NaNi_0.3_Fe_0.2_Mn_0.5_O_2_	Interface/transport	107 mAh g^–1^ (2.0–4.0 V) and 125 mAh g^–1^ (2.0–4.2 V) at 10 C	Multi-step synthesis	[83]
Na_2_Ti_3_O_7_ (residual-alkali-derived)	O3-Na_0.95_Ni_1/3_Fe_1/3_Mn_1/3_O_2_	Residual alkali/wettability	101.9 mAh g^−1^ at 10 C; 85.66% over 200 cycles at 1 C	Ti-source-dependent uniformity	[58]
DHP self-assembled monolayer	O3-NaNi_0.33_Fe_0.33_Mn_0.33_O_2_	Air/moisture	87.3% over 330 cycles at 1 C; 118 mAh g^−1^ after 7-day humid air	Organic-layer long-term stability	[84]
Buffered fluorination (Na_3_FeF_6_)	O3-Na(NiFeMn)_1/3_O_2_	Fe dissolution/O3′ phase	84.7% retention over 1000 cycles at 2 C	Humid-process control	[85]
Ionic impedance-matched interphase (Na_3_PO_4_/Ti-spinel)	P2-Na_5/6_Li_1/4_Mn_3/4_O_2_	Oxygen redox/interface	~230 mAh g^−1^; <0.05 V voltage decay	Multi-step conversion	[75]
Core–shell (Mg-rich P2 shell)	P2-Na_0.67_[Ni_0.25_Mn_0.75_]O_2_ core	P2→O2 transition	81% retention over 1000 cycles at 5 C	Sharp core–shell boundary	[77]
Mg-gradient shell (surface gradient desodiation)	Na_0.72_Ni_0.20_Co_0.21_Mn_0.55_Mg_0.036_O_2_	TM migration/rock-salt	3000 cycles; 0.127 mV cycle^−1^ decay	Coprecipitation control	[78]
High-entropy lattice pinning	O3-Na_0.96_Ca_0.02_Ni_0.27_Fe_0.2_Mn_0.35_ Al_0.02_Cu_0.1_Ti_0.05_O_2_	Phase transition/air	1.1% strain; 80.1% over 1000 cycles at 5 C; 264.87 mAh g^−1^ (full cell)	Dilutes capacity; cost	[43]
High-entropy (interlayer-surface regulation)	NaNi_0.3_Mn_0.3_Fe_0.2_Ti_0.1_Cu_0.06_ Mg_0.04_O_1.95_F_0.05_	Air/phase transition	130.8 mAh g^−1^ at 0.1 C; 85% over 500 cycles at 2 C; 88.6% after 30-day air	Multi-element synthesis	[86]
DMDF co-solvent (NaF-rich CEI)	O3-Na_0.85_Mn_0.45_Ni_0.25_Li_0.05_Cu_0.1_Ti_0.15_O_2_	Solvent oxidation/CEI	80% over 600 cycles; 304.5 Wh kg ^−1^ (full cell)	Additive cost	[81]
Ternary solvent (SL/DMC/PFPN)	NaNi_1/3_Fe_1/3_Mn_1/3_O_2_	CEI/oxidation	85.72% over 300 cycles at 3 C; CEI modulus 19.97 GPa	Fluorinated-solvent cost	[80]
Li/Ti/Co co-doping + AlPO_4_ coating	O3-NaNi_1/3_Fe_1/3_Mn_1/3_O_2_	Phase transition + interface	45.6% → 83.6% over 100 cycles at 1 C; 0.08 V decay	Multi-step	[87]

**Table 2:** Failure cascade, dominant length scale, and the modulation strategies that interrupt each link.

Cascade Link	Dominant Length Scale	Interrupting Strategies	Cited Examples
Residual alkali/air instability	atomic	Residual-alkali conversion, hydrophobic SAM, Na-free surface	[34,41,47,58,74]
Lattice-oxygen loss	atomic	TM–O covalency, redox-mediator (Fe), dielectric coating	[25,27,29,31,82]
Slab gliding/phase transition	atomic–nano	Pillar ions, lattice-coherent intergrowth, high-entropy	[40,43,48]
Surface reconstruction/rock-salt	nano	Conformal/lattice-matched/ion-conducting coating	[41,58,75]
TM dissolution/HF corrosion	nano	HF-scavenging/fluorinated interface, residual-alkali conversion	[44,70,85]
Intergranular cracking/strain	micro	Single crystal, gradient/core–shell, isotropic morphology	[45,77,78]
CEI instability/cross-electrode crosstalk	cell	Electrolyte/CEI engineering, additive/co-solvent design	[80,81,104,110]

## Data Availability

The original contributions presented in this study are included in the article. Further inquiries can be directed to the corresponding authors.
